# Veterinary antimicrobials practices for swine mycoplasmoses: a field survey and comparison with scientific and regulatory recommendations

**DOI:** 10.1186/s40813-026-00522-4

**Published:** 2026-06-22

**Authors:** Romain Hollard, Anneke Feberwee, Salvatore Catania, Katarzyna Dudek, Jade Bokma, Lucía Manso-Silván, Glenn Browning, Dominiek Maes, Anne V. Gautier-Bouchardon, Manon Holstege, Florence Tardy, Jeanine Wiegel, Claire A.M. Becker

**Affiliations:** 1 Université de Lyon, VetAgro Sup, Marcy L’Etoile, 69280 France; 2 Royal GD, Deventer, The Netherlands; 3 Mycoplasma Unit, WOAH Reference Laboratory for Avian Mycoplasmosis, SCT-1 Verona, Istituto Zooprofilattico Sperimentale delle Venezie, Via Bovolino 1c, 37060 Buttapietra, (VR) Italy; 4 Department of Bacteriology and Bacterial Animal Diseases, National Veterinary Research Institute, 57 Partyzantów Avenue, Pulawy, 24-100 Poland; 5 Department of Internal Medicine, Reproduction and Population Medicine, Faculty of Veterinary Medicine, Ghent University, Salisburylaan 133, Merelbeke, B-9820 Belgium; 6 CIRAD INRAE, UMR ASTRE, Montpellier, F-34398 France; 7 Asia-Pacific Centre for Animal Health, Melbourne Veterinary School, Faculty of Science, The University of Melbourne, Victoria, 3010 Australia; 8 ANSES, Laboratoire de Ploufragan-Plouzané-Niort, Unité Mycoplasmologie, Bactériologie et Antibiorésistance, Ploufragan, France; 9 Université de Lyon, UMR Mycoplasmoses Animales, ANSES, VetAgro Sup, Marcy L’Etoile, 69280 France; 10 Veterinary Practice Venhei, Kasterlee, 2400 Belgium

**Keywords:** Swine, Mycoplasmas, Antimicrobial use, Veterinarian, Survey

## Abstract

Mycoplasma infections pose a significant challenge in swine production because of their economic impact and the difficulties associated with diagnosis and treatment. Antimicrobials are often the only available option for combating mycoplasmoses despite reports of resistance to several molecules and the frequent chronic nature of infections, which can reduce treatment efficacy. This study aimed to investigate the use of antimicrobials against swine mycoplasmoses through an international field survey and to review the relevant scientific literature and regulatory recommendations. The survey was led by the MyMIC network, whose main objective is to standardise antimicrobial susceptibility testing (AST) methods for livestock mycoplasmas. The responses of 118 swine veterinarians across 25 countries were analysed. No significant variations in practices were observed between countries. Macrolides, tetracyclines, and pleuromutilins were the most prescribed antimicrobial classes (used in more than 40%, 20% and 5% of clinical cases, respectively), which is consistent with current recommendations. Additional diagnosis with laboratory-based methods was commonly reported, with only 2.5% of veterinarians indicating that they never used it. However, AST was not commonly performed; only 30% of respondents reported requesting it in more than half of the cases. Ineffective or critically important antibiotics, such as beta-lactams or fluoroquinolones, were occasionally prescribed (approximately 1% for each). Occasional deviations from the recommended dosages were also noted. These findings raise concerns regarding resistance selection and potential treatment failures. Our results underscore the need for enhanced diagnosis, especially AST, and for a wider dissemination and adoption of treatment guidelines to support the sustainable management of swine health. By developing and promoting harmonised AST protocols and enabling objective, data-driven prescriptions, MyMIC plays a key role in strengthening communication between swine veterinarians and diagnostic laboratories, thereby supporting international antimicrobial stewardship.

**Supplementary Information:**

The online version contains supplementary material available at https://doi.org/10.1186/s40813-026-00522-4.

## Background

Mycoplasmas are a major cause of disease in pigs and represent a significant source of economic losses. As such, they are a major concern for the effective management of swine health [1].

Several *Mycoplasma* species are pathogenic in pigs, with *Mycoplasma hyopneumoniae* (*M. hyopneumoniae*), *Mycoplasma hyorhinis* (*M. hyorhinis*), *Mycoplasma hyosynoviae* (*M. hyosynoviae*) and *Mycoplasma suis* (*M. suis*) being the most significant. Regarding respiratory diseases, the main pathogenic species is *M. hyopneumoniae* (*M. hyopneumoniae*), which is the primary pathogen of enzootic pneumonia (EP) and contributes to the porcine respiratory disease complex (PRDC) [2]. *M. hyopneumoniae* can infect pigs at any age [2]. It adheres to the ciliated cells of the respiratory epithelium, invades host cells, and secretes cytotoxic proteins, all of which contributes to impaired mucociliary clearance [3]. Infected pigs can have a dry cough, respiratory distress, reduced average daily weight gain (ADWG) and a higher feed conversion ratio. Gross lesions range from marked pulmonary oedema in acute infections to diffuse parenchymal discolouration in chronic cases [4]. *M. hyorhinis* is also commonly detected in the respiratory tract of pigs, as well as a culture contaminant, and it is frequently found in human carcinomas [5]. In pigs, it may cause polyarthritis and polyserositis and may also be involved in pneumonia, otitis, and conjunctivitis [6, 7]. Gross lesions include pericarditis, pleuritis, peritonitis, and arthritis [8]. Finally, *M. hyosynoviae* infection is also associated with arthritis, mainly in fattening pigs; however, the factors that determine the development of clinical disease following infection are still unclear [9]. *M. suis* may affect erythrocytes and cause anaemia in pigs [2]. Other *Mycoplasma* species, including *Mycoplasma flocculare* and *Mycoplasma hyopharyngis*, can also infect pigs but are considered opportunistic pathogens potentially involved in multifactorial disease processes [10, 11].

In diagnostic laboratories, mycoplasmas detection relies primarily on molecular methods, particularly PCR, because culture of these bacteria may be technically challenging and time consuming [12]. The detection of *M. hyopneumoniae* is of particular concern, as it is regularly overgrown by *M. hyorhinis*. Culture in an acellular medium is not yet possible for *M. suis*. Regarding indirect diagnosis, many serological tests are poorly specific, and ELISA tests are preferentially used to detect antibody responses against infections with *M. hyopneumoniae* at the group level [2, 13–22]. To control mycoplasmoses in pigs, there are currently no effective commercial vaccines against *M. hyorhinis*, *M. hyosynoviae,* or *M. suis* and the protection provided by vaccines against *M. hyopneumoniae* is only partial [23]. In this context, control mainly relies on antimicrobial treatments and good health management practices. While studies have been conducted to determine which antimicrobials are effective against swine mycoplasmas in vitro and what are the acquired resistances [7], the actual use of antimicrobials by veterinarians to specifically treat mycoplasmoses (clinical and subclinical) in the field is however not known.

This study was conducted within the framework of the MyMIC network, funded by the Joint Programming Initiative on Antimicrobial Resistance (JPIAMR) [24], and the main goal of MyMIC is to standardise culture, identification, and antimicrobial susceptibility testing (AST) methodologies for *Mycoplasma* species of veterinary importance [12], to develop recommendations for the surveillance and management of mycoplasma infections in farm animals (poultry, pigs and ruminants). The objective of the present work was to obtain an overview of field practices for the control of swine mycoplasmoses, with particular emphasis on antimicrobial use (first-choice treatment, metaphylactic approaches), as well as the methods employed by diagnostic laboratories and veterinarians to detect infection and inform treatment decisions. This information was obtained through a survey of livestock veterinarians inside and outside Europe. This paper reports and discusses the results of the questionnaire sent to veterinarians involved in pig production, in the context of existing scientific and regulatory guidelines.

## Methods

### Survey design

A survey was designed to collect data about the antimicrobials used by veterinarians worldwide to treat mycoplasma infections in multiple livestock species (cattle, poultry and swine) [12]. It comprised 23 questions covering the methods veterinarians use to diagnose mycoplasma infections; their preferred treatment approaches, including first-choice antimicrobial drugs; the alternatives used in case of treatment failure; their approach to the metaphylactic use of antimicrobials; and the reasons behind these choices. The questionnaire design was based on a previous survey developed in 2022 to assess the underlying reasons for the ongoing use of colistin in livestock [25]. This survey was adapted in order to collect information about the diagnosis and treatment of mycoplasma infections in multiple livestock species. This article presents the results related to swine only, using the terms of the survey. Details on the survey are available in a previous article [12]. The webpage layout and the pig questionnaire are available in the supplementary materials (Supp. Fig. 1).

The survey could be completed anonymously; however, veterinarians were given the option to provide their e-mail address at the end of the questionnaire if they wished to be informed of the results.

### Data handling and statistical analyses

Once the questionnaire was closed, the data were exported from Formdesk® [26] into a Microsoft® Excel file and then split into a different Microsoft® Excel file for each animal species. Calculations of percentages and graphical analyses were conducted via R [27].

Three tables are presented in this study. Table 1 shows the number of veterinarians following or not following the recommended doses and durations for the treatment of mycoplasma infections, by antimicrobial class and pig category. To generate this table, the doses and treatment durations prescribed by veterinarians were compared with the Summary of Product Characteristics (SmPC) of the molecules available in their respective country of practice. SmPCs were collected from multiple websites across different countries [28–36].

**Table 1:**
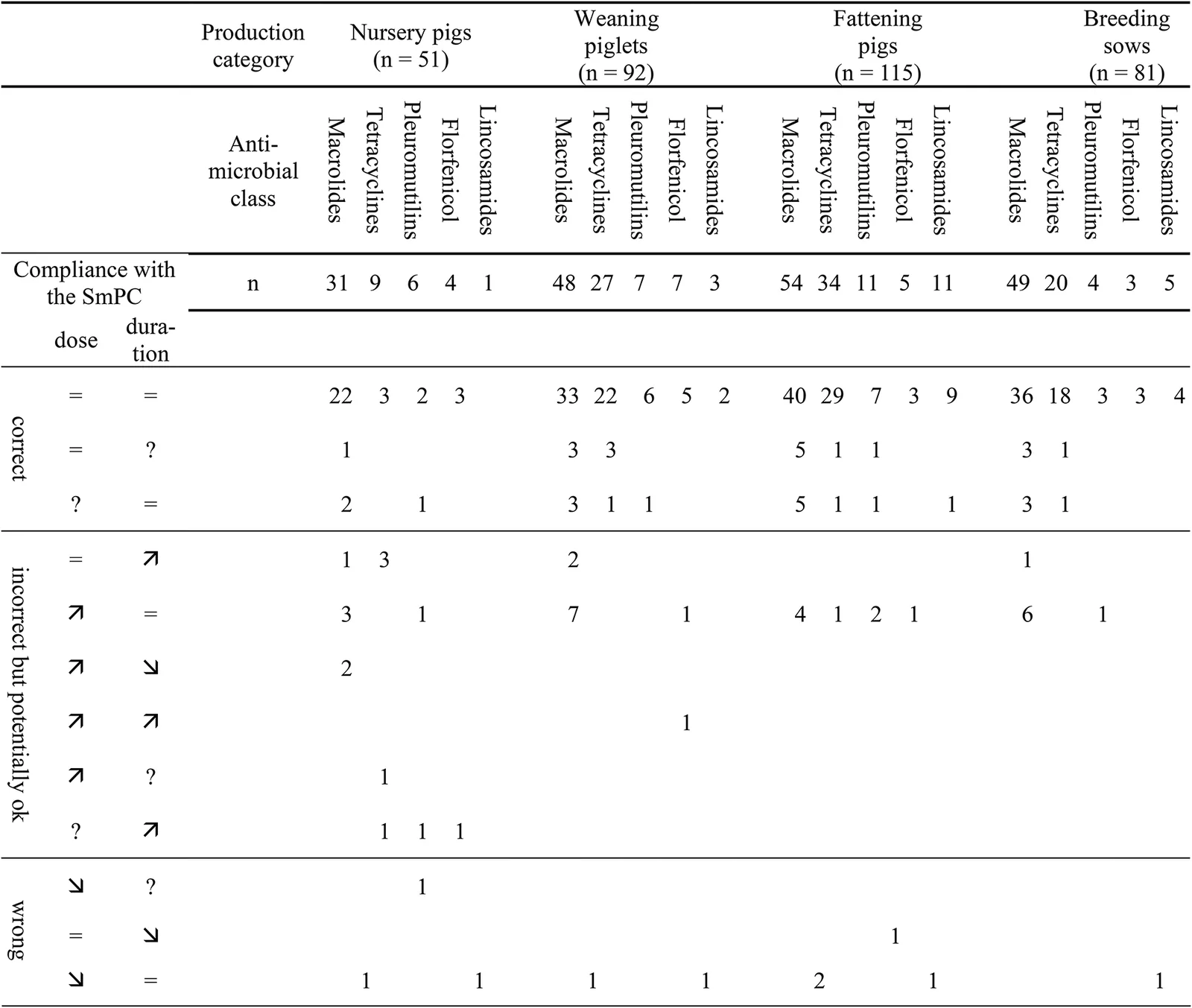
Number of responses of veterinarians per dose and duration for each production category and antimicrobial class (*n* = 116)

Table 2 lists the antimicrobials that are expected to be effective against the three main *Mycoplasma* species affecting pigs based on in vitro AST data currently reported in the literature.

**Table 2:** Overview of the antimicrobials for each Mycoplasma species for which in vitro susceptibility was reported

	M. hyopneumoniae	M. hyorhinis	M. hyosynoviae
Aminoglycosides		Gentamicin, Spectinomycin [37, 38]	Gentamicin, Spectinomycin [39, 40]
Phenicols	Florfenicol [41]	Florfenicol [37, 42]	Florfenicol [40]
Fluoroquinolones	Enrofloxacin, Marbofloxacin [41, 43, 44]	Enrofloxacin [37, 38, 42, 45]	Enrofloxacin, Danofloxacin [39, 40, 45, 46]
Lincosamides	Lincomycin [41]	Lincomycin [38, 47]	Lincomycin, Clindamycin [40, 46]
Macrolides	Spiramycin, Tulathromycin, Gamithromycin, Tylosin, Tilmicosin, Tylvalosin [41, 43, 47]	[38, 42, 45, 47]	[40, 45–47]
Pleuromutilins (Tiamulin, Valnemulin)	[41, 43, 44, 47]	[37, 42, 45, 47]	[39, 40, 45, 46]
Tetracyclines	Doxycycline, Oxytetracycline [41]	Doxycycline, Oxytetracycline, Chlortetracycline [37, 38, 42, 45]	Oxytetracycline, Chlortetracycline [40, 45, 46]

Legend: Antimicrobial classes are sorted alphabetically; all studies referenced are in vitro studies based on MIC determination

Table 3 shows the antimicrobial classes recommended for the treatment of infections with each of the three main *Mycoplasma* species affecting pigs according to published guidelines from national veterinary associations, supported or not supported by the government, Health Ministers and veterinarian specialists of these countries: Switzerland [48], Belgium [49], Germany [50], Sweden [51], Australia [52], New Zealand [53], Canada [54], the United Kingdom [55], the Netherlands [56] and Italy [57].

**Table 3:** National guidelines for the treatment and control of mycoplasma infections in pigs

*Guidelines provided by*	*National Veterinary Associations*						*National Veterinary Associations supported by the Government*					*Health Ministry*	*Vet. Special.*
*Country*	Germany*	*Belgium*	*Netherlands¤*		*New Zealand*	*Sweden*	*Australia*	*Canada*		*Switzerland*		*Italy*	*United Kingdom*
			*Oral*	*Parenteral*				*Oral*	*Parenteral*	*Oral*	*Parenteral*		
*Mhyo*		**Prev.**			Prev.			**Prev.**	**Prev.**				
		Gent.											
		Spec.		Spec.(2)									
								Flor.	Flor.		Flor.		
	LINs.	Linco.	Linco.	Linco.(2)			Linco.	*Linco*.	*Linco.*			LINs.	Linco.
	FQs.	*Enr.*				*Enro.*							
		*Marb.*			*Marb.*								
		Tilm.	Tilm.				Tilm.						
	MACs	Tylo.	Tylo.	**Tylo.(1)**	*Tylo.*		Tylo.	*Tylo*.	*Tylo.*	*Tylo.*	*Tylo.*	*MACs*	*MACs*
		Tylva.	Tylva.										
		Tul.		Tul.(1)			Tul.	*Tul.*					
	PLEUs.		**Tia.**	**Tia.(2)**	** Tia.**	PLEUs.	**Tia.**	Tia.	Tia.	Tia.	*Tia.*	**PLEUs.**	**PLEUs.**
			**Val.**										
	TETs.	Oxyt.	Oxyt.°	**Oxyt.(3)**	**TETs.**	**TETs.**		TETs.	TETs.	**Doxy.**	**Oxyt.**	**TETs.**	TETs.
		Doxy.	*Doxy.*										
*Mhr*		ND	ND			ND			ND	Arthritis:	Arthritis:	ND	ND
										Tylo.	Tylo.		
					Linco.			Linco.		**Tia**.	**Tia.**		
										*Doxy.*	*Oxyt.*		
					*Tylo.*			*Tylo.*					
										Polyser.:	Polyser.:		
											*Flor.*		
					**Tia.**			**Tia.**			FQs.°		
											Tylo.°		
					**TETs.**			**TETs.**		**Tia.**	**Tia.**		
										Doxy.	Oxyt.		
										*Chlort.*			
*Mhs*		ND	ND		ND			Linco.	ND			ND	Linco.
								Tylo.		Tylo.	Tylo.		
								**Tia.**		**Tia.**	**Tia.**		**Tia.**
						**TETs.**		**TETs.**		*Doxy.*	*Oxyt.*		

Legend: *Mhyo*, M. hyopneumoniae; *Mhr*, M. hyorhinis; *Mhs*, M. hyosynoviae; LINs., Lincosamides; Linco., Lincomycin; FQs., Fluoroquinolones; Enro., Enrofloxacin; Marb., Marbofloxacin; PLEUs., Pleuromutilins; Tia., Tiamulin; Val., Valnemumlin; TETs., Tetracyclines; Oxyt., oxytetracycline; Doxy., Doxycycline; MACs, Macrolides; Tilm., Tilmicosin; Tylo. Tylosin; Tylva., Tylvalosin; Tul., Tulathromycin; Gent., Gentamicin; Spec., Spectinomycin; Polyser., Polyserositis; Prev., preventive measures, such as vaccination and improved environmental management; Vet. Special., Veterinarian Specialists; ND, No data

Antimicrobial drugs shown in **bold** are the recommended first-choice treatments, those shown underlined are the recommended second-choice treatments, those shown in italic text are the recommended third-choice treatments, and those shown with (°) are the recommended fourth-choice treatment. These guidelines were found in the following references (36-45). *The German guidelines provide a list of antimicrobials for the treatment of *Mycoplasma * infections, whatever the species. ¤The Netherlands guidelines provide two levels of choice, the first one in bold and the second one underlined; for each level the order of preference are indicated in brackets

## Results

Among the 468 veterinarians who responded to the whole survey [12], 134 (28.6%) reported treating pigs in 25 countries. Only their responses addressing general considerations or pig-related questions are analysed in this study.

### Demographic features of respondent swine veterinarians

The greatest number of responses were from France (*n* = 55), followed by Germany (*n* = 11), South Africa (*n* = 10), Italy (*n* = 9), Canada (*n* = 8), Austria (*n* = 7), Australia (*n* = 5), Argentina and the Netherlands (*n* = 4 each), Belgium and Hungary (*n* = 3 each), and the United Kingdom (*n* = 2). The remaining responses (*n* = 1 each) were from Burkina Faso, the Czech Republic, Finland, Ireland, Kenya, Mexico, Norway, Pakistan, the Philippines, Spain, Sweden, Switzerland, and the United States.

Because the number of responses was too small, comparison between European countries, between continents, or between other categories such as veterinary experience or specialisation were not possible. Results regarding some specific questions (metaphylaxis, treatment choice and duration, and diagnostics) are provided per country in additional tables included as supplementary data. The analysis was performed on the full set of 134 surveys, with particular focus on the 118 respondents who reported dealing with mycoplasma infections in pigs.

More than one-third of the responding livestock veterinarians had over 25 years of experience as practitioners (*n* = 50), followed by those with 6–15 years (*n* = 36), 16–25 years (*n* = 35), and fewer than five years of experience (*n* = 13). The largest proportion of respondents had one to three livestock veterinarians in their practice (*n* = 38), while similar numbers worked in practices with four to six (*n* = 32), seven to nine (*n* = 32), or more than ten (*n* = 32) livestock practitioners. Most of the respondents worked with nursery piglets, weaning piglets, fattening pigs, and breeding sows (*n* = 116, 117, 122, and 120, respectively). In addition to pigs, they mentioned working with layer chickens, breeder chickens, broiler chickens, and meat turkeys (*n* = 24, 21, 25, and 16, respectively) and, finally, dairy cattle, beef cattle, and cattle rearing (*n* = 23, 26, and 19, respectively).

### Most frequently encountered *Mycoplasma* species

In response to the question “Do you encounter *Mycoplasma* spp. infections in the animals you see?”, the vast majority of veterinarians answered “yes” (*n* = 127, 94.8%), with 118 responses specifically for pigs. The following analyses were done on these 118 responses. The veterinarians reported that the swine production categories most affected by mycoplasmoses were fattening pigs (*n* = 115), weaning piglets (*n* = 84), breeding sows (*n* = 69), and nursery pigs (*n* = 44).

**Fig. 1:**
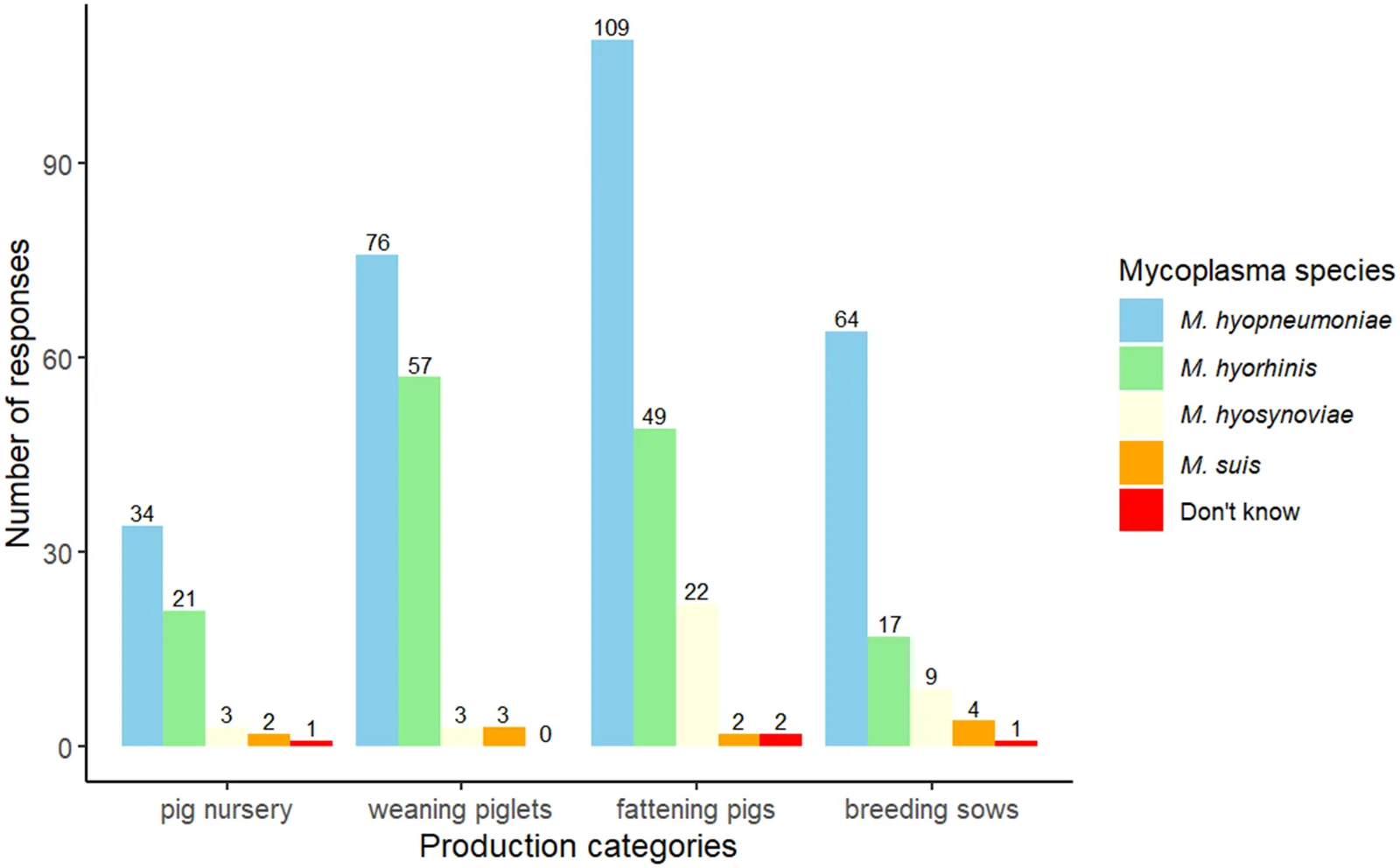
Mycoplasma species associated with clinical disease detected by veterinarians per swine production category (*n* = 118). Legend: numbers of responses to the multiple-choice question “what Mycoplasma species do you encounter related to clinical diseases?” are presented per production category. The veterinarians could answer for each production category they worked with. There was a possibility to propose other species, hence the addition of M. hyosynoviae and M. suis

Veterinarians were asked which *Mycoplasma* species was (presumably) associated with the clinical disease they encountered. The options for this multiple-choice question were ‘*M. hyopneumoniae’*, ‘*M. hyorhinis’*, ‘Other’ and ‘Don’t know’. The results are shown in Fig. 1. *M. hyosynoviae* and *M. suis* were added by veterinarians. In more than 50% of the cases, the *Mycoplasma* species associated with each production category was *M. hyopneumoniae*, followed by *M. hyorhinis*, *M. hyosynoviae*, and *M suis*.

Among veterinarians who reported encountering clinical mycoplasma infections in swine production (*n* = 118), the largest proportion indicated that 10–25% of animals were affected (*n* = 46). This was followed by estimates of 25–50% (*n* = 28), more than 50% (*n* = 23), and less than 10% (*n* = 21).

### Diagnostic approach and frequency of AST

**Fig. 2:**
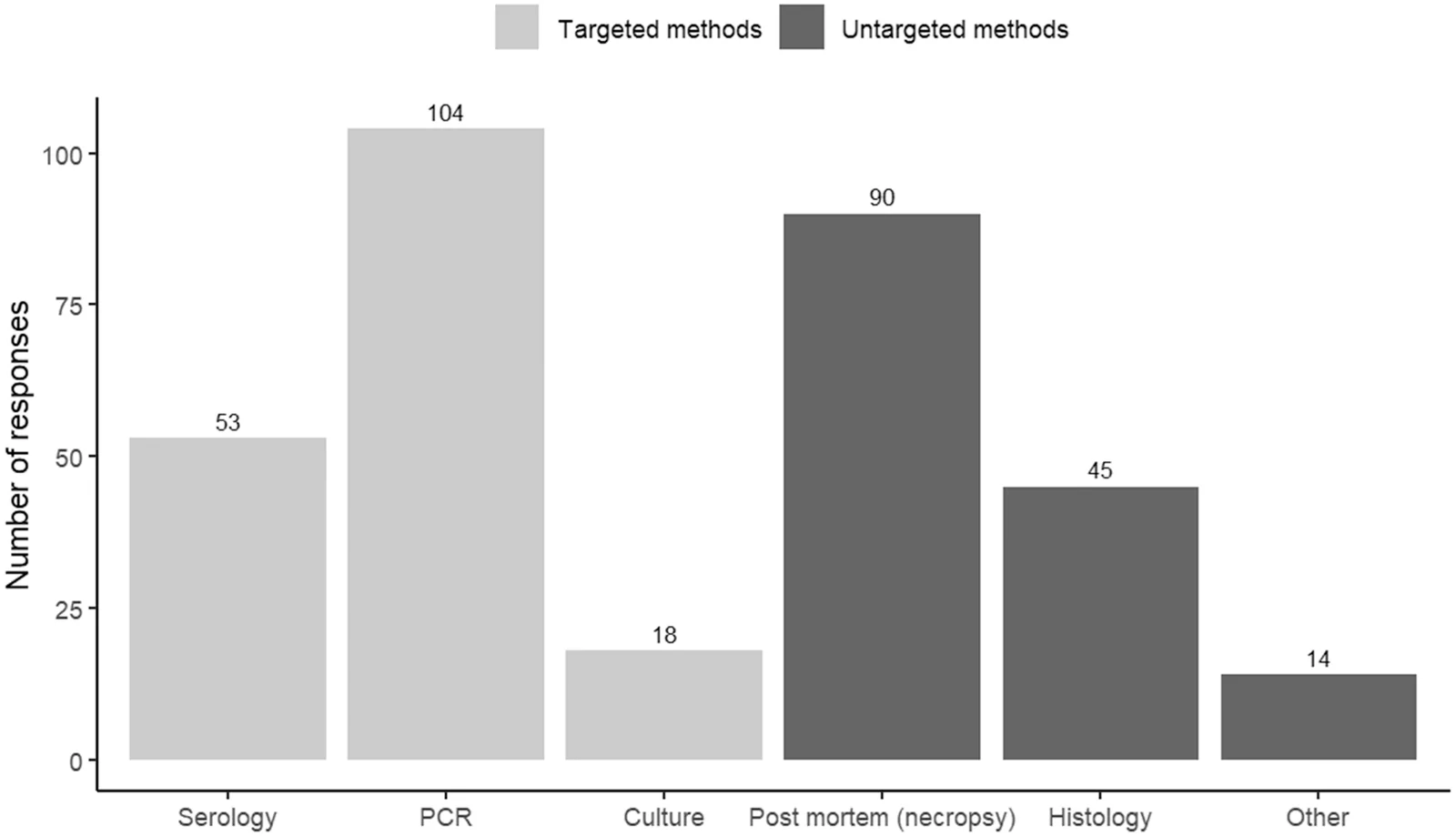
Additional diagnostic methods applied by veterinarians/laboratories when a mycoplasma infection was suspected (*n* = 115). Legend: number of responses selected for each option in the multiple-choice survey question “if yes, what additional diagnosis?” is represented. The first three methods (serology, PCR and culture) are specific methods, whereas the remaining ones (necropsy, histology and the other responses reported) are conclusive about the disease but cannot confirm the aetiological pathogen

Almost all the veterinarians requested additional diagnosis when a mycoplasma infection was suspected on the basis of clinical signs, with 26% always using laboratory-based diagnosis (*n* = 31), 27% using it often (more than 50% of the time) (*n* = 32), 27% using it occasionally (between 10 and 50% of the time) (*n* = 32), 17% using it rarely (less than 10% of the time) (*n* = 20), and only 2.5% never using it (*n* = 3). As shown in Fig. 2, the additional diagnostic methods used were most commonly PCR and necropsies, followed by serology and histology. Of the fourteen ‘Other’ responses, eleven veterinarians cited ‘slaughterhouse checks’, which could be viewed as resembling ‘Postmortem (necropsy)’, although the limited inspection of the respiratory tract provides substantially less information. PCR, culture and serology are methods that specifically target mycoplasmas, whereas the other methods are nonspecific but can support a diagnosis of the disease.

However, when a mycoplasma infection was confirmed, 61% of veterinarians never or rarely (less than 10% of cases) requested ASTs from the isolates (*n* = 54 and 18 respectively). This response was predominant (more than 50% of respondents) in all parts of the world, as for example in France, Canada, South Africa, Australia or the Netherlands. On the contrary, only 30% (*n* = 35) of veterinarians often or always (more than 50% of the time) requested AST; the countries where AST was frequently requested (more than 50% of the respondents) were Germany (*n* = 6/11), Italy (*n* = 6/9), Belgium (*n* = 2/3) and the United States (*n* = 1/1).

### Preferred treatment options

Nearly all the veterinarians indicated that treatment was required for mycoplasma infections in pigs, with only one of 118 respondents stating that treatment was ‘never’ necessary. Sixteen indicated that treatment was ‘always’ required, while 55 indicated that it was ‘often’ required (in more than 50% of cases). Among the list of options proposed, the main reasons chosen by the veterinarians for applying treatment to pigs with mycoplasmoses were, in order of importance, the severity of clinical signs (*n* = 103/117), the proportion of animals showing clinical signs (*n* = 69/117), the results of laboratory tests (*n* = 47/117), the perception that this is a “disease which spreads quickly” (*n* = 31/117), and, less commonly, that treatment was “requested by farmers or the industry” (*n* = 9/117).

**Fig. 3:**
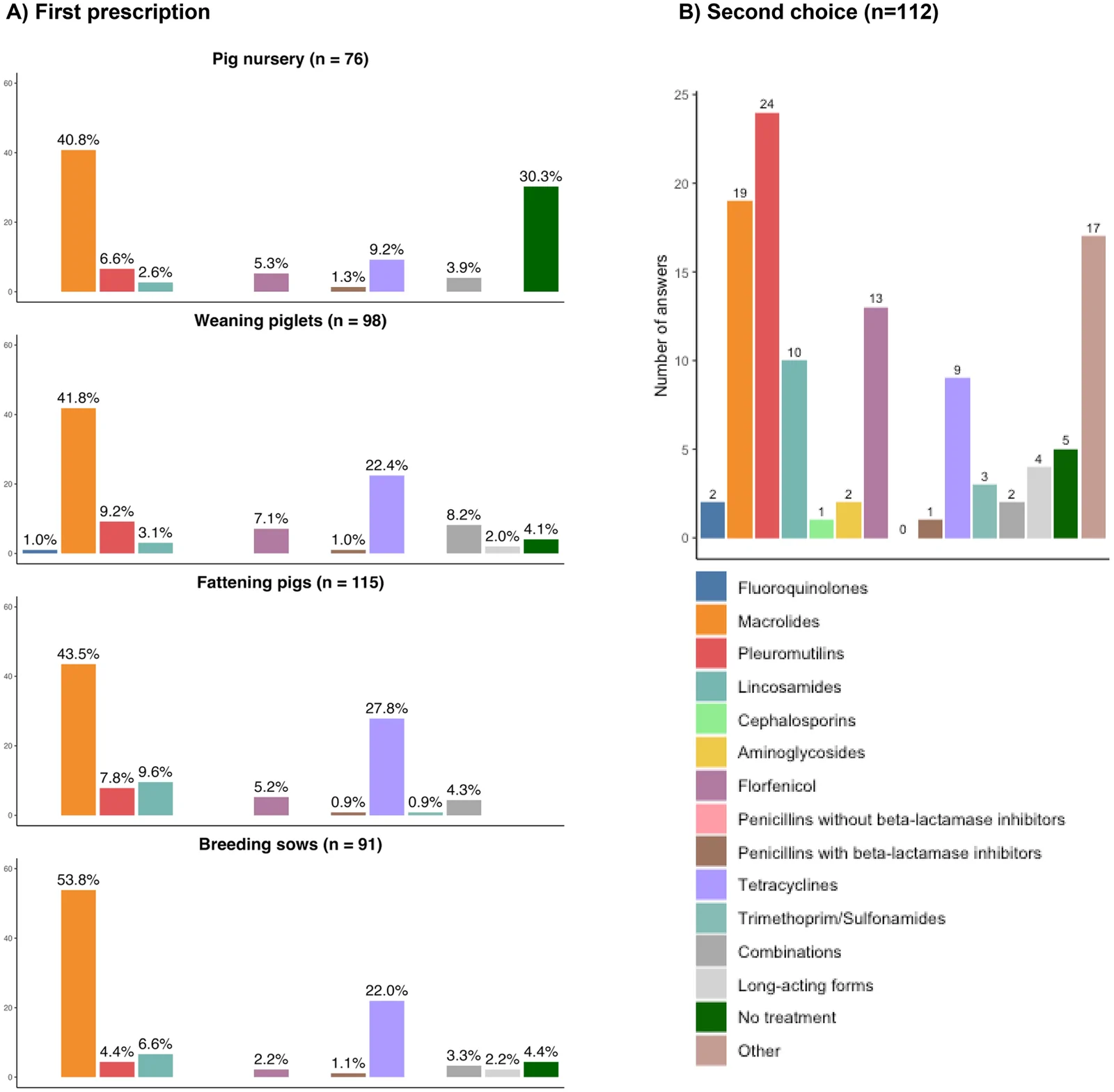
Preferred antimicrobial class chosen for the first treatment of pigs with mycoplasma infections in each production category (left part) and selected as a second choice in cases of treatment failure (*n* = 112). Legend: A; the percentage of total responses to the question “what is the preferred option of treatment?” is represented per production category. The number of responses per category varies according to the number of veterinarians treating these categories. B; the number of responses to the question “in case of failure of treatment, what is the second choice of treatment available?” is represented

The preferred options for treating mycoplasmoses are shown in Fig. 3. Across all the production categories, most mycoplasma infections were treated with macrolides, tetracyclines, pleuromutilins, lincosamides, or florfenicol. The same antimicrobial classes could be used in combination or in long-acting formulations. Four out of eight veterinarians who reported sometimes using beta-lactams indicated that their treatment was aimed at secondary infections.

For both questions, veterinarians could only select one antimicrobial family.

The preferred treatment options were selected based on national treatment guidelines (*n* = 52/118), known antimicrobial susceptibility profiles of the pathogen (*n* = 50/118), farm history (*n* = 41/118), or the economic feasibility (*n* = 25/118) and AST results (*n* = 20/118).

A total of 116 veterinarians also provided indications on the doses and treatment durations for their preferred options in each production category (Table 1). Most veterinarians adhered to the SmPC recommendations when using the drug (67% for pig nurseries, 86% for weaning piglets, 90% for fattening pigs, and 89% for breeding sows). Most veterinarians indicated that they used the recommended doses and duration of treatment (except for pig nurseries), particularly for macrolides and tetracyclines. However, deviations to recommendations were reported, including the use of higher doses (*n* = 31), longer treatments (*n* = 11), lower doses (*n* = 9), or shorter treatments (*n* = 3).

Among the 102 veterinarians who occasionally chose not to treat mycoplasma infections, the main reasons for withholding treatment were withdrawal periods (*n* = 37), treatment costs (*n* = 32), legal boundaries (*n* = 27), expected lack of efficacy (*n* = 16), and restrictions due to trade requirements or industry agreements (*n* = 10). Of the 36 veterinarians who provided “other” reasons, 16 indicated a preference for using vaccination to control mycoplasmoses instead.

### Second-choice for treatment in the event of initial treatment failure

Among the 117 veterinarians who reported the use of antimicrobials to treat mycoplasmoses in pigs, 112 provided information on their second-choice treatment in the event of initial treatment failure (Fig. 3B). Pleuromutilins were the antimicrobial class most frequently selected as second-choice option, followed by macrolides, florfenicol, lincosamides, and tetracyclines. The combinations and long-acting formulations used contained only macrolides, and those that already used them as first-choice reported switching to a different molecule. Some veterinarians mentioned in the ‘Other’ section that first-choice treatment failure (most often a macrolide) was uncommon (*n* = 12/17) and that, in the event of treatment failure, they preferred not to administer a second treatment.

### Use of metaphylaxis

When asked about metaphylaxis, 18% (*n* = 21) of the respondents reported never using this strategy to control mycoplasmoses in pigs. Another 25% (*n* = 30) stated that they used it rarely (less than 10% of the time), 30% (*n* = 35) used it occasionally (between 10 and 50% of the time), 21% (*n* = 25) often used it (more than 50% of the time), and only 6% (*n* = 7) always used metaphylactic medication for managing mycoplasmoses in pigs. The rationale of the 97 veterinarians who used metaphylaxis was as follows: the severity of clinical signs (*n* = 67); the proportion of animals with clinical signs (*n* = 58); the presence of “known disease that spreads quickly” (*n* = 40); the results of laboratory tests (*n* = 27); and, for a few respondents, “requested by farmers or the industry” (*n* = 9). The most common patterns of metaphylactic medication used by swine veterinarians for each antimicrobial class are shown in Supp. Figure 2. Macrolides, tetracyclines, pleuromutilins and lincosamides are predominantly administered in the drinking water, whereas florfenicol and fluoroquinolones are primarily administered by intramuscular injection. Combinations of macrolides and tetracyclines are exclusively administered *per os* (mainly in the drinking water), whereas long-acting forms (macrolides, tetracyclines and florfenicol) are mostly administered via intramuscular injection.

### Scientific and national guidelines

Table 2 summarises the antimicrobials reported as potentially active based on published Minimal Inhibitory Concentration (MIC) data for each *Mycoplasma* species, which represent the unique data currently available in swine. Neither clinical breakpoints nor epidemiological cut-offs (ECOFFs) are currently available. VetCAST reports only a single MIC distribution for *M. hyopneumoniae* with florfenicol [58], which is insufficient to define an ECOFF. A key prerequisite for establishing clinical breakpoints and ECOFFs is the availability of a standardised consensual AST MIC methodology [12]. Table 3 presents the national guidelines published by animal health authorities or veterinary associations from Switzerland, Belgium, Germany, Sweden, Australia, New Zealand, Canada, the United Kingdom, the Netherlands, and Italy. Most veterinarians followed the scientific and national guidelines by predominantly using macrolides, tetracyclines, florfenicol, lincosamides, and pleuromutilins for first- and second-choice treatments.

## Discussion

### Prescriptions and consumption data

This survey provides an overview of practices regarding diagnostic testing and antimicrobial use for mycoplasma infections in pigs across various countries worldwide. The diversity in respondent demographics, including years of experience, type and number of veterinarians in the practices, etc. ensures a broad panel of perspectives. However, it is important to note that a significant proportion of the respondents (41%) were from France, which may have introduced some bias. Nonetheless, the varied backgrounds of respondents make this a valuable overview of the approaches used to control mycoplasma infections in pigs. The antimicrobial drugs most frequently prescribed by veterinarians for first-choice treatment of mycoplasma infections were macrolides, tetracyclines, pleuromutilins, lincosamides, and florfenicol, for both individual treatment and metaphylaxis. According to surveillance reports on European sales and use of antimicrobials for veterinary medicine, published by the European Medicines Agency (EMA) covering the period from 2010 to 2024 [59–61], penicillins were the most commonly sold class of antimicrobials for production animals. Data on antimicrobial use remained partial, as a 90% coverage was achieved for only 12 countries in 2024 (excluding France), representing approximately 50% of the total European pig biomass [61]. Among the available data, penicillins were the most frequently used antimicrobials, followed by tetracyclines, then macrolides. In contrast to these general data, the responses in our study clearly indicate an appropriate adjustment of therapeutic practices by veterinarians when dealing with mycoplasmosis, in a manner consistent with the known microbiological characteristics of these atypical pathogens.

### Comparison between field data and recommendations

According to the literature, major swine *Mycoplasma* species are generally susceptible in vitro to macrolides, tetracyclines, pleuromutilins, lincosamides, fluoroquinolones, florfenicol, and certain aminoglycosides (Table 2) [7]. This spectrum aligns with national recommendations found worldwide for the treatment of mycoplasmoses in pigs, despite a few minor variations between countries. The consensual list across countries includes pleuromutilins, tetracyclines, macrolides, and lincosamides, with the use of fluoroquinolones usually only regarded as a last resort or not permitted in food-producing animals, such as in Australia (Table 3).

The antimicrobial classes most frequently cited by the veterinarians in the survey as first-choice treatments for mycoplasmoses in pigs (Fig. 3) are similar to those recommended in the national guidelines: macrolides, tetracyclines, pleuromutilins, and, less frequently, lincosamides and florfenicol. The recruitment of volunteers to participate in the survey may have introduced selection bias. Indeed, the veterinarians who chose to participate were likely those with a particular interest in mycoplasma infections and/or with better awareness of appropriate antimicrobial use for these specific bacteria. The treatment practices reported by the veterinarians completing the survey were generally consistent with the recommendations provided in national antimicrobial use guidelines. For example, macrolides, which are widely recommended internationally (Table 3) for the treatment of *M. hyopneumoniae* infections, were the antimicrobials most frequently prescribed by respondents (Fig. 3). Similarly, pleuromutilins and tetracyclines were also commonly prescribed in accordance with most national treatment guidelines. The only exception was a minority of responses mentioning the use of beta-lactams, including for first-choice treatment, for mycoplasmoses without further explanation. This raises serious concerns about some veterinarians having very poor knowledge of mycoplasma specificities, as these classes have no efficacy against mycoplasmas. The present results are entirely based on veterinarians’ self-reports. As these studies can be subject to recall bias, social desirability bias (a tendency to report practices perceived as “correct”), and overestimation of the actual frequency of certain practices, it is possible that the results do not fully reflect the reality in the field. To gain a more accurate view of antimicrobial use for mycoplasmoses worldwide, sales data need to be analysed by animal species or production categories, and not aggregated for all livestock per country (as in the last 3 reports of the EMA, from 2010 to 2024 [59–61]). Moreover, not only sales but also the use reported by veterinarians themselves should be studied. This has been gathered by the EMA for 2 years, however with incomplete data for many countries [60, 61]. This highlights the importance of online systems such as Calypso Vet [62] in France or SANITEL-MED [63] in Belgium, which are new tools capable of recording all antimicrobial prescriptions issued by veterinarians. Some of these online systems, such as the Danish antimicrobial use surveillance system (DANMAP) [64], collect detailed data on use for different body systems and classes of animals that may enable a specific focus on major mycoplasma diseases. However, this information is currently not available in other countries. In the future, combining data from various national systems would help provide a clearer picture of which antimicrobials are used in specific contexts.

Several differences were noted regarding the use of fluoroquinolones. Although this antimicrobial class is listed as a therapeutic option in some national guidelines (Germany, Belgium, New Zealand, Sweden, and Switzerland), it is generally recommended only as a last-resort treatment. Fluoroquinolones are classified as critically important [65], and their use is regulated in Europe and restricted as a first choice treatment (category B) by the EMA [66]. Regulations recommend that their use be cautious, reserved for situations where alternative therapies have failed, and justified by AST results. In our survey, fluoroquinolones were occasionally listed by respondents as first- and second-choice treatments. Additional insights from veterinarians are needed to clarify the reasons for their use as first-line therapy. Since 61% of the respondents in our survey reported never or rarely requesting AST, much of the use of fluoroquinolones for treating mycoplasmoses may be empirical, which contradicts the principles of appropriate antimicrobial use. Nevertheless, the application of this regulation is a challenge when *Mycoplasma* spp. are involved, as most veterinary laboratories do not offer AST for these bacteria. This suggests that national policies to preserve critical antimicrobials should be implemented more rigorously and that the accessibility to AST for pathogenic porcine mycoplasmas should be improved. In addition, professional training on the concept of MICs and the appropriate use of antimicrobials should be strengthened and more widely implemented to generate objective data to support antimicrobial stewardship decisions. Notably, fluoroquinolones were not commonly used by the veterinarians who responded to our survey, a positive finding that indicates prudent prescribing practices and limited use of this critical antimicrobial class in the field.

### Identification of practices that may contribute to antimicrobial resistance in mycoplasmas

#### Mycoplasma diagnostic and AST challenges

There were inconsistencies in the practices applied by some veterinarians to diagnose mycoplasma infections in pigs. As shown in Fig. 1, most veterinarians indicated that they could reliably identify the *Mycoplasma* species causing clinical disease on a farm. Indeed, the ‘Don’t know’ responses constitute a minority regardless of the production category. Most respondents reported that in over 50% of cases, they used additional diagnostic methods to confirm their suspicion of mycoplasmoses, despite the fact that the cited methods could not provide a definitive etiologic diagnosis. Furthermore, PCR was the most commonly used method, while culture was used infrequently (15.6%, Fig. 2), despite being necessary for subsequent AST. Even if molecular methods are common for diagnosis, their use for AMR determination is promising at this stage for mycoplasmas but culture and AST remain currently the only reliable methods [67].

AST was rarely performed, with only 30% of veterinarians reporting that they had requested it in the majority of cases where mycoplasmas were diagnosed. If we consider only respondents that asked for diagnosis by culture (*n* = 18), 44.4% (*n* = 8) requested AST in more than half the cases (often and always). These findings suggest that even when mycoplasma infections are confirmed by culture, MICs testing for the causative organism of various antimicrobials is rarely performed. This is to be expected, as culture and AST are time-consuming, so treatment is often initiated without AST results. However, knowledge of AST remains important for field practice, as the availability of population-based surveillance MIC data can contribute to improving treatment strategies and preventing inappropriate or ineffective antimicrobial use and consequently treatment failure [68]. In addition, global surveillance of antimicrobial resistance in mycoplasmas is essential, and veterinarians play a key role in this effort by requesting AST when appropriate.

The limited use of AST in mycoplasma infections can be attributed to several factors, such as the challenges associated with the isolation of these particular organisms, the lack of standardised methods for AST, the time needed to obtain MICs for mycoplasmas, and the limited access to specialised laboratories capable of isolating and testing the antimicrobial susceptibility of porcine mycoplasmas [12]. AST request was reported by most of the veterinarians only in four countries (Germany, Italy, Belgium and the United States of America), showing discrepancies between countries. Even if the number of respondents was very low for these countries (ranging from 1 to 11), these results highlight the fact that AST may not be widely available. Thus, treatment is often initiated on the basis of species identification and the veterinarian’s experience, without assessment of antimicrobial susceptibility, even though all pathogenic porcine mycoplasmas can develop acquired resistance to the antimicrobial drugs commonly used for the treatment of the diseases they cause, especially macrolides, tetracyclines and lincosamides [39]. This highlights also the need to develop harmonised AST methods for swine mycoplasmas, as well as interpretative criteria linked to MIC, and to increase the availability of these methods worldwide for laboratories as well as for veterinarians.

#### Treatment practices that may promote antimicrobial resistance in mycoplasmas

Several antimicrobial treatment strategies for porcine mycoplasmoses have been identified that may compromise treatment efficacy and promote antimicrobial resistance.

First, some respondents reported using antimicrobials (beta-lactams, trimethoprim and sulfonamides) that are known to be ineffective against mycoplasmoses. Mycoplasmas lack a cell wall and thus are inherently resistant to beta-lactams and other antimicrobials that target peptidoglycan biosynthesis [39]. They also lack the folate biosynthesis pathway and thus are not affected by trimethoprim or sulfonamides. However, some practitioners (*n* = 8 in Fig. 3A and *n* = 1 in Fig. 3B) reported using penicillin with beta-lactamase inhibitors for first- or second-choice treatment for mycoplasma infections without mentioning another reason for this treatment (*n* = 4). This suggests that some veterinarians may not be aware that mycoplasmas are not susceptible to these antimicrobial drugs or that their therapy focused on other bacterial organisms contributing to the PRDC. Some practitioners using penicillins indicated that they focused on the treatment of coinfections with other bacteria (*n* = 4). However, mycoplasmas can be the sole pathogen responsible for or a major contributor to PRDC, so it is important to consider treatments that target both the mycoplasmas and the coinfecting pathogens. While it is important to reduce the burden of coinfecting bacteria, there is no need to use beta-lactams, as these bacteria can also be susceptible to other antimicrobials effective against mycoplasmas [7].

Suboptimal doses and treatment durations were also reported. Doses lower than those recommended by the SmPC were reported for several classes of antibiotics (Table 1, *n* = 8). Underdosing not only compromises clinical efficacy but also contributes to the selection of resistant strains [69]. Similarly, shorter treatment durations than those recommended were also reported by one respondent (Table 1). This shorter treatment, although accompanied by the administration of increased doses (Table 1, *n* = 2), may increase the risk of clinical relapse and the development of resistance [70]. The impact of other practices that are not compliant with current recommendations, including overdosing or increasing the duration of treatment, is less certain than underdosing. However, the precautions for safe use mentioned in the SmPCs for antimicrobials specify that the recommended doses and treatment times should be respected. In general, guidelines do not distinguish between overdosing and underdosing consequences. Furthermore, excessive use of antimicrobials increases costs of treatment for the farmer (direct treatment cost and longer withdrawal period) and the global consumption of antimicrobials, thereby increasing the risk of selection of resistance in pigs and the environment.

Metaphylactic medication was also commonly used to control mycoplasmoses; it was reported by 57% of respondents for more than 10% of the cases. No differences were observed between European countries versus the other parts of the world (data not shown). While metaphylactic medication may be justified for the control of an outbreak with high morbidity, it remains controversial if applied without evidence-based herd indicators (e.g. % of ill animals in the flock). The efficacy of metaphylactic medication in controlling mycoplasma infections during the fattening phase is limited [71]. Mass medication via drinking water or feed, the primary route of administration for macrolides, tetracyclines, lincosamides, and pleuromutilins, should be guided by a careful risk-benefit assessment and combined with other control measures - such as improved hygiene and vaccination- as suggested by some survey respondents and recommended in the literature, particularly for mycoplasmoses [7].

Some veterinarians reported that they did not choose a second-line treatment if the first treatment failed (*n* = 12). This decision may have been influenced by an emphasis on limiting antimicrobial use or by the possibility that viral infections played a primary role in the PRDC [72, 73]. This practice may still contribute to the selection of resistant strains and facilitate the persistence of infections involving copathogens. The persistence of infection or clinical signs after treatment failure should at least be balanced by other control methods or be followed up by diagnostic reassessment and reconsideration of the need for, or the choice of, therapy and should ideally be guided by AST. Otherwise, not only does the clinical problem persist, but the risk of resistance selection (both in mycoplasmas and other copathogens) is potentially increased.

### Limitations and strengths of the study

#### Limitations

Survey-based studies have inherent limitations. In this study, responses may have been biased towards veterinarians familiar with mycoplasmoses, limiting insight into the practices of those less aware of these diseases. In addition, the broadly framed questions, designed to apply across the three livestock categories (including cattle and poultry), may not have fully captured field realities or may have been subject to misinterpretation.

Mycoplasmoses in pigs can be due to different mycoplasma species, with *M. hyopneumoniae* being the most important. However, it is difficult to know which mycoplasmas the veterinarians had in mind when completing the questionnaire.

The population of respondents was heterogeneous and biased in favour of France; the numbers of responses per country were not sufficient to perform further statistical analyses, but it was not our aim. As pig raising can be very different between continents and farms (small-scale farms versus large integrated production systems), interpretations could be made only from a general point of view.

#### Strengths

This study is the first attempt to gather information on antimicrobial use against swine mycoplasmoses, as well as diagnostic strategies.

The questionnaire was based on a survey that had previously been proven effective [25]. It was adapted to the context of mycoplasmoses by the authors as experts in the field.

The international consortium MyMIC has managed to reach the veterinary population across the world. Therefore, a rather large number (absolute value) of participants was achieved.

An important achievement of this work is highlighting the need for more uniform recommendations owing to the heterogeneity between countries. The MyMIC network will play a crucial role in achieving this goal from a policy perspective.

Importantly, this project has initiated the first dialogue between mycoplasmologists and veterinarians. Sharing these findings with professionals, such as at scientific conferences, will help maintain this dialogue, which is crucial for enhancing antimicrobial stewardship.

## Conclusion

Our international survey regarding diagnostic and treatment strategies for the control of mycoplasmoses in pigs revealed the use of macrolides, tetracyclines and pleuromutilins as first-choice treatments. Most of the treatment practices reported by the respondents were consistent with international scientific recommendations and national guidelines for antimicrobial use, indicating that the majority of veterinarians are able to manage mycoplasmoses appropriately. However, the relatively infrequent use of AST, the continued use of critically important antimicrobials for treating mycoplasmoses, and deviations from recommended doses and treatment durations may contribute to the selection of resistant strains of pathogenic porcine mycoplasmas or other pathogens, increasing the risk of treatment failure.

These risks underscore the importance of disseminating updated recommendations for effective control and developing reliable, accessible diagnostic methods - particularly for AST- to optimise antimicrobial use in swine production, thereby improving livestock health management and combating antimicrobial resistance.

The routine use of MIC testing for mycoplasma species remains limited, due to logistical constraints, the scarcity of specialised laboratories, and prolonged turnaround times. First-line therapy for these pathogens primarily relies on veterinarians’ clinical experience, while MIC testing is generally reserved for second-line therapeutic reassessments. Moving forward, it is crucial to establish data-driven therapy at the farm level. MIC results should be used to inform future therapeutic protocols within specific farms or integrated production chains, aligning with Regulation (EU) 2019/6 (specifically Recital 45 and Article 105), which emphasises antimicrobial susceptibility testing (AST) and requires veterinarians to justify prescriptions. Ultimately, mycoplasmologists and diagnostic laboratories must strive together to make MIC testing more accessible for these fastidious microorganisms across different regions. Concurrently, Competent Authorities should play a pivotal role in promoting antimicrobial stewardship –including mycoplasmas- based on objective data, including national surveillance and trends in susceptibility patterns over time.

## Electronic supplementary material

Below is the link to the electronic supplementary material.

Supplementary Material 1

Supplementary Material 2

## Data Availability

The responses of the survey and the analysed datasets obtained during the current study are available from the corresponding author upon reasonable request.

## References

[1] Kobisch M, Marois C. Les mycoplasmoses porcines. Bulletin de l'Académie Vétérinaire de France. 2008;161(2):179–84. 10.4267/2042/47941.

[2] Maes D, Sibila M, Pieters M. Mycoplasmas in swine. Wallingford, UK: CABI; 2020.

[3] Leal Zimmer FMA, Paes JA, Zaha A, Ferreira HB. Pathogenicity & virulence of *Mycoplasma hyopneumoniae*. Virulence. 2020;11(1):1600–22. 10.1080/21505594.2020.1842659. 2020-12-31. PMC7733983 33289597

[4] Zimmerman JJ, Karriker LA, Ramirez A, Schwartz KJ, Stevenson GW. Diseases of swine. 10th. Hoboken, New Jersey, USA: Wiley-Blackwell; 2012.

[5] Huang S. Mycoplasma infections and different human carcinomas. WJG. 2001;7(2):266–69. 10.3748/wjg.v7.i2.266. 11819772 PMC4723534

[6] Lefèvre P-C, Blancou J, Chermette R, Uilenberg G. Infectious and parasitic diseases of livestock. Cachan, France: Tec & Doc Lavoisier; 2010.

[7] Maes D, Boyen F, Haesebrouck F, Gautier-Bouchardon AV. Antimicrobial treatment of *Mycoplasma hyopneumoniae* infections. The Vet J. 2020;259-260:05/2020;259–260. 105474. 10.1016/j.tvjl.2020.105474. 32553237

[8] Kobisch M, Friis NF. Swine mycoplasmoses. Revue Scientifique et technique (international Office of Epizootics). 1996. Revue scientifique et technique (Int Office Epizootics). 199612;15(4):1569–605. 10.20506/rst.15.4.983. 9190026

[9] Nielsen EO, Nielsen NC, Friis NF. *Mycoplasma hyosynoviae* Arthritis in grower-finisher pigs. J Vet Med Ser A. 2001;48(8):475–86. 10.1046/j.1439-0442.2001.00378.x. 2001-10-9. 11710673

[10] Földi D, Nagy EZ, Tóth G, Makrai L, Gombos L, Kreizinger Z, et al. *Mycoplasma hyopharyngis* isolated from the joint of a weaner: a case report. AVet. 2024;72(3):155–60. 10.1556/004.2024.01078. 2024-09-12. 39213125

[11] Fourour S, Tocqueville V, Paboeuf F, Lediguerher G, Morin N, Kempf I, et al. Pathogenicity study of *Mycoplasma hyorhinis* and *M. flocculare* in specific-pathogen-free pigs pre-infected with *M. hyopneumoniae*. Vet Microbiol. 2019;232:50–57. 10.1016/j.vetmic.2019.04.010. 2019-05-1. 31030844

[12] Jaÿ M, Klose SM, Bottinelli M, Autio T, Becker CAM, Bokma J, et al. Advancing standardization of diagnostics and antimicrobial susceptibility testing for pathogenic mycoplasmas of livestock origin: insights from the MyMIC network. BMC Vet Res. 2025, 2025/12/29; 21(1):712. 10.1186/s12917-025-05154-4. 41462247 PMC12751987

[13] Bai Y, Gan Y, Hua L-Z, Nathues H, Yang H, Wei Y-N, et al. Application of a sIgA-ELISA method for differentiation of *Mycoplasma hyopneumoniae* infected from vaccinated pigs. Vet Microbiol. 2018;223:86–92. 10.1016/j.vetmic.2018.07.023. 30173757

[14] Burrough E, Schwartz A, Gauger P, Harmon K, Krull A, Schwartz K. Comparison of postmortem airway swabs and lung tissue for detection of common porcine respiratory pathogens by bacterial culture and polymerase chain reaction assays. J Swine Health Prod. 2018;26(5):246–52. 10.54846/jshap/1092. 2018-09-1.

[15] Fablet C, Marois C, Kobisch M, Madec F, Rose N. Estimation of the sensitivity of four sampling methods for *Mycoplasma hyopneumoniae* detection in live pigs using a Bayesian approach. Vet Microbiol. 2010;143(2–4):238–45. 20036079 10.1016/j.vetmic.2009.12.001

[16] Friis NF. [Mycoplasms of the swine--A review]. Nord Vet Med. 1975;27(6):329–36. 1153279

[17] Giménez-Lirola LG, Meiroz-De-Souza-Almeida H, Magtoto RL, McDaniel AJ, Merodio MM, Matias Ferreyra FS, et al. Early detection and differential serodiagnosis of *Mycoplasma hyorhinis* and *Mycoplasma hyosynoviae* infections under experimental conditions. PLoS One. 2019;14(10):e0223459. 10.1371/journal.pone.0223459. 2019-10-7. PMC6779295 31589633

[18] Kurth KT, Hsu T, Snook ER, Thacker EL, Thacker BJ, Minion FC. Use of a *Mycoplasma Hyopneumoniae* nested polymerase chain reaction test to determine the optimal sampling sites in swine. J Vet Diagn Invest. 2002;14(6):463–69. 10.1177/104063870201400603. 12423027

[19] Pieters M, Daniels J, Rovira A. Comparison of sample types and diagnostic methods for in vivo detection of *Mycoplasma hyopneumoniae* during early stages of infection. Vet Microbiol. 2017;203:103–09. 10.1016/j.vetmic.2017.02.014. 28619131

[20] Roos LR, Surendran Nair M, Rendahl AK, Pieters M. *Mycoplasma hyorhinis* and *Mycoplasma hyosynoviae* dual detection patterns in dams and piglets. PLoS One. 2019;14(1):e0209975. 2019-1-3. 10.1371/journal.pone.0209975 PMC6317828 30605453

[21] Sponheim A, Alvarez J, Fano E, Schmaling E, Dee S, Hanson D, et al. Comparison of the sensitivity of laryngeal swabs and deep tracheal catheters for detection of *Mycoplasma hyopneumoniae* in experimentally and naturally infected pigs early and late after infection. Vet Microbiol. 2020;241:108500. 10.1016/j.vetmic.2019.108500. 31767388

[22] Vangroenweghe F, Karriker L, Main R, Christianson E, Marsteller T, Hammen K, et al. Assessment of litter prevalence of *Mycoplasma hyopneumoniae* in preweaned piglets utilizing an antemortem tracheobronchial mucus collection technique and a real-time polymerase chain reaction assay. J Vet Diagn Invest. 2015;27(5):606–10. 10.1177/1040638715595062. 26179099

[23] Maes D, Segales J, Meyns T, Sibila M, Pieters M, Haesebrouck F. Control of *Mycoplasma hyopneumoniae* infections in pigs. Vet Microbiol. 2008;126(4):297–309. 10.1016/j.vetmic.2007.09.008. 17964089 PMC7130725

[24] MyMIC -JPIAMR. 2025. https://jpiamr.eu/projects/mymic/

[25] Jansen W, Van Hout J, Wiegel J, Iatridou D, Chantziaras I, De Briyne N. Colistin use in European livestock: veterinary field data on trends and perspectives for further reduction. Vet Sci. 2022;9(11):650. 10.3390/vetsci9110650. 2022-11-21. 36423099 PMC9697203

[26] Formdesk, Wassenaar, the Netherlands 2024. https://formdesk.com/en/about-formdesk/

[27] Team RC. R: a language and environment for Statistical Computing. Vienna, Austria: R Foundation for Statistical Computing; 2024.

[28] Institut für Veterinärpharmakologie und toxikologie. Tierarzneimittel (Schweiz). Universität Zürich. 2025 [updated 2025]; Available from: https://www.vetpharm.uzh.ch/perldocs/kompend3.htm

[29] Australian Pesticides and Veterinary Medicines Authority (APVMA). Public chemical registration information System search. 2025 [updated 2025]; Available from: https://portal.apvma.gov.au/pubcris

[30] European Medicines Agency (EMA). European sales and use of antimicrobials for veterinary medicine (EsuaVet). Annual surveillance report for 2023. Luxembourg2025; (EMA/CVMP/ESUAVET/80289/2025)]. Available from: https://medicines.health.europa.eu/veterinary/en

[31] US Food and Drug Administration (FDA). Animal Drugs @ FDA. 2025. [updated 2025]; Available from: https://animaldrugsatfda.fda.gov/adafda/views/#/search.

[32] Finnish Medicines Agency (Fimea). FMA FimeaWeb - Fimea - YJA. 2025 [updated 2025]; Available from: https://fimea.fi/en/databases_and_registers/fimeaweb

[33] Government of Canada. Drug product database: access the database. Transparency - Other;education and awareness. 2010 [updated 2010-03-18]; Available from: https://www.canada.ca/en/health-canada/services/drugs-health-products/drugproducts/drug-product-database.html

[34] South African Health Products Regulatory Authority (SAHPRA). List of registered veterinary products. 2025 [updated 2025]; Available from: https://www.sahpra.org.za/list-of-registered-veterinary-product/

[35] ServicioNacional de Sanidad y Calidad Agroalimentaria (Senasa). Registro de productos veterinarios. Dirección de Tecnología de la Información, Argentina. 2025. [updated 2025]; Available from https://aps2.senasa.gov.ar/vademecumVet/app/publico/farmacos

[36] Veterinary Medicines Directorate. Product information database. Government of United Kingdom. 2025 [updated 2025]; Available from: https://www.vmd.defra.gov.uk/productinformationdatabase/search

[37] Bekő K, Felde O, Sulyok KM, Kreizinger Z, Hrivnák V, Kiss K, et al. Antibiotic susceptibility profiles of *Mycoplasma hyorhinis* strains isolated from swine in Hungary. Vet Microbiol. 2019;228:196–201. 10.1016/j.vetmic.2018.11.027. 2019-01-1. 30593367

[38] Wu CC, Shryock TR, Lin TL, Faderan M, Veenhuizen MF. Antimicrobial susceptibility of *Mycoplasma hyorhinis*. Vet Microbiol. 2000;76(1):25–30. 10.1016/s0378-1135(00)00221-2. 10925038

[39] Gautier-Bouchardon AV. Antimicrobial resistance in *Mycoplasma* spp. Microbiol. Spectr. 2018;6(4):6.4.07. 2018-07-27. 10.1128/microbiolspec.arba-0030-2018 PMC11633602 30003864

[40] Schultz KK, Strait EL, Erickson BZ, Levy N. Optimization of an antibiotic sensitivity assay for *Mycoplasma hyosynoviae* and susceptibility profiles of field isolates from 1997 to 2011. Vet Microbiol. 2012;158(1–2):104–08. 22397937 10.1016/j.vetmic.2012.02.002

[41] De Jong A, Youala M, Klein U, El Garch F, Moyaert H, Simjee S, et al. Antimicrobial susceptibility monitoring of *Mycoplasma hyopneumoniae* isolated from seven European countries during 2015–2016. Vet Microbiol. 2021;253:108973. 10.1016/j.vetmic.2020.108973. 33418394

[42] Klein U, Földi D, Belecz N, Hrivnák V, Somogyi Z, Gastaldelli M, et al. Antimicrobial susceptibility profiles of *Mycoplasma hyorhinis* strains isolated from five European countries between 2019 and 2021. PLoS One. 2022;17(8):e0272903. 10.1371/journal.pone.0272903. 2022-8-11. PMC9371350 35951622

[43] Gonzaga NF, De Souza LFL, Santos MR, Assao VS, Rycroft A, Deeney AS, et al. Antimicrobial susceptibility and genetic profile of *Mycoplasma hyopneumoniae* isolates from Brazil. Braz J Microbiol. 2020;51(1):377–84. 31797326 10.1007/s42770-019-00185-0 PMC7058719

[44] Hannan PCT, Windsor HM, Ripley PH. In vitro susceptibilities of recent field isolates of Mycoplasma hyopneumoniae and Mycoplasma hyosynoviae to valnemulin (econor), tiamulin and enrofloxacin and the in vitro development of resistance to certain antimicrobial agents in Mycoplasma hyopneumoniae. Res Vet Sci. 1997;63(2):157–60. 10.1016/s0034-5288(97)90010-2. 9429250

[45] Kobayashi H, Sonmez N, Morozumi T, Mitani K, Ito N, Shiono H, et al. In vitro susceptibility of *Mycoplasma hyosynoviae* and *M. hyorhinis* to antimicrobial agents. 1996;58(11). 10.1292/jvms.58.11_1107 8959659

[46] Aarestrup FM, Friis NF. Antimicrobial susceptibility testing of *Mycoplasma hyosynoviae* isolated from pigs during 1968 to 1971 and during 1995 and 1996. Vet Microbiol. 1998. 10.1016/s0378-1135(98)00169-2 9646463

[47] Rosales RS, Ramírez AS, Tavío MM, Poveda C, Poveda JB. Antimicrobial susceptibility profiles of porcine mycoplasmas isolated from samples collected in southern Europe. BMC Vet Res. 2020;16(1):324. 10.1186/s12917-020-02512-2. 32883288 PMC7469352

[48] Faculté Vetsuisse, Société des Vétérinaires Suisses (SVS), Office fédéral de la sécurité alimentaire et des affaires vétérinaires (OFAV). Utilisation prudente des antibiotiques: Bovins, Porcs, Petits Ruminants et Camélidés du Nouveau Monde Guide thérapeutique pour les vétérinaires. Mars. 202

[49] AntiMicrobial Consumption and Resistance in Animals (Amcra). AMCRA Formularium FR. 2024 [updated 2024]; Available from: https://formularium.amcra.be/a/3

[50] Bundestierärztekammer (BTK). Guidelines for the prudent use of veterinary antimicrobial drugs - with notes for guidance -. Germany2015 January 2015.

[51] Lindberg M. Guidelines for the use of antibiotics in production animals - cattle, pigs, sheep and goats. Sweden: The Swedish Veterinary Association (SVA) Swedish Veterinary Society (SVS)2013.

[52] Australian Veterinary Association (AVA), Animal Medicines Australia (AMA), Australian Departmentof Agriculture and Water Resources through the Australian Biosecurity Responseand Reform Program (ABRR), Australian Pork Limited (APL). Antimicrobial prescribing guidelines for pigs. Australia2020. 2020

[53] New Zealand Veterinary Association (NZVA). Guide to prudent use of antimicrobial agents in pigs. New Zealand2020. 2020, Jan.

[54] Canadian Veterinary Medical Association, Agriculture and Agri-Food Canada. Canadian veterinary medical Association antimicrobial prudent use guidelines 2008 for beef cattle, dairy cattle, poultry, and swine. Canada2008. 2008

[55] Burch DGS, Duran CO, Aarestrup FM. Guidelines for antimicrobial use in swine. In: Guardabassi L, Jensen LB, Kruse H, editors. Guide to antimicrobial Use in animals. 1. Blackwell Publishing, Ltd; 2008. p. 102–25.

[56] van Duijkeren E, van Hout Aj, den Hartog Pa, Mjf K, van Nes A, Mar S. FORMULARIUM VARKEN. Neth: KONINKLIJKE NEDERLANDSE MAATSCHAPPIJ VOOR DIERGENEESKUNDE (KNMVD), WERKGROEP VETERINAIR ANTIBIOTICUMBELEID2019 semptember 2019.

[57] Patrizia B, Chiara C, Simone L, Andrea L, Deborah M, Giuseppe M, et al. Linee guida - Uso prudente degli antibiotici nell’allevamento suino. Italy: Ministero della Salute2025 2025.

[58] Testing ECoAS. Data from the EUCAST MIC distribution website. 2025 [cited 2025, 22/12/2025]; Available from: https://mic.eucast.org/search/?search%5Bmethod%5D=mic%26search%5Bantibiotic%5D=-1%26search%5Bspecies%5D=344%26search%5Bdisk_content%5D=-1%26search%5Blimit%5D=50.

[59] European Medicines Agency (EMA). Sales of veterinary antimicrobial agents in 31 European countries in 2022. Trends from 2010 to 2022. Luxembourg: Publications Office of the European Union; 2023.

[60] European Medicines Agency (EMA). European sales and use of antimicrobials for veterinary medicine (ESUAvet). Annual surveillance report for 2023. 2025. Luxembourg: Publications Office of the European Union.

[61] European Medicines Agency (EMA). European sales and use of antimicrobials for veterinary medicine (ESUAvet). Annual surveillance report for 2024. 2025. Luxembourg: Publications Office of the European Union.

[62] Ordre National des Vétérinaires. Calypso: un guichet unique au service des vétérinaires | L’Ordre national des vétérinaires. 2022 [updated 2022-07-05]; Available from https://www.veterinaire.fr/la-profession-veterinaire/nos-grands-dossiers/calypso-un-guichet-unique-au-service-des-veterinaires

[63] Agence fédérale des médicaments et des produits de santé (AFMPS). SANITEL-MED | AFMPS. 2025 [updated 14/01/2025]; Available from: https://www.afmps.be/fr/SANITEL-MED

[64] Statens Serum Institut. The Danish integrated antimicrobial resistance monitoring and Research programme (DANMAP). 2025 [updated 2025]; Available from: https://www.danmap.org/9725793

[65] Scott HM, Acuff G, Bergeron G, Bourassa MW, Gill J, Graham DW, et al. Critically important antibiotics: criteria and approaches for measuring and reducing their use in food animal agriculture. Ann N Y Acad Sci. 2019;1441(1):8–16. 10.1111/nyas.14058. 30924540 PMC6850619

[66] European Medicines Agency (EMA). Categorisation of antibiotics in the European Union. Answer to the request from the European Commission for updating the scientific advice on the impact on public health and animal health of the use of antibiotics in animals. Luxembourg: Publications Office of the European Union; 2025.

[67] Sulyok KM, Kreizinger Z, Földi D, Kovács ÁB, Grózner D, Manso-Silván L, et al. Molecular detection of antimicrobial resistance in livestock mycoplasmas: current status and future prospects. Front Vet Sci. [Review]. 2025;2025–December-15;Volume 12–2025. 10.3389/fvets.2025.1699077 PMC12745486 41473103

[68] Jaÿ M, Poumarat F, Colin A, Tricot A, Tardy F. Addressing the antimicrobial resistance of ruminant mycoplasmas using a clinical surveillance network. Frontiers in veterinary Science. Original Res. 2021;8(667175). 10.3389/fvets.2021.667175 PMC8236625 34195247

[69] Roberts JA, Kruger P, Paterson DL, Lipman J. Antibiotic resistance-What’s dosing got to do with it? Crit Care Med. 2008;36(8). 10.1097/CCM.0b013e318180fe62 18596628

[70] Xia X, Yang L, Ling Y, Yu J, Ding H. Emergence and mechanism of resistance of Tulathromycin against *Mycoplasma hyopneumoniae* in a PK/PD Model and the fitness costs of 23S rRNA mutants. Front Vet Sci. 2022;9:801800. 10.3389/fvets.2022.801800. 2022-2-11. 35224081 PMC8873822

[71] Ramirez CR, Harding AL, Forteguerri EBR, Aldridge BM, Lowe JF. Limited efficacy of antimicrobial metaphylaxis in finishing pigs: a randomized clinical trial. Preventative Vet Med. 2015;121(1–2):176–78. 10.1016/j.prevetmed.2015.06.002. 26130504

[72] Arruda BL, Derscheid RJ. Respiratory System. Chapter 21. In: Jeffrey Zimmerman EB, Karriker LOCKE, Schwartz KENT, Zhang JIANQIANG, editors. Diseases of swine. 12th. Hoboken, New Jersey: John Wiley & Sons, Inc; 2025. p. 439–56.

[73] Carr J, Sibila M, Segalés J. *Mycoplasma hyopneumoniae*: clinical signs and gross lung lesions, including monitoring. Chapter 11. In: Maes D, Sibila M, Pieters M, editors. Mycoplasmas in Swine: acco. 2020. p. 96–107.

