# Supplementary material for: Veterinary antimicrobials practices for swine mycoplasmoses: a field survey and comparison with scientific and regulatory recommendations
Source: Porcine Health Manag. 2026 Jun 22;12:50. doi: 10.1186/s40813-026-00522-4 (PMC13595520; doi:10.1186/s40813-026-00522-4)
Supplement: Supplementary file 2 [file 40813_2026_522_MOESM2_ESM.docx]

*Supplementary Figure 2: Patterns of metaphylactic treatment most frequently used by pig veterinarians for each antimicrobial classes*

Legend: This figure represents the answers to the multiple-choice question: “what is the most frequent pattern for metaphylactic treatment that you apply in your practice?”. The respondents had to tick the corresponding boxes (antimicrobial and administration route); they could answer for each family. The percentages are calculated on the numbers of answers for each category (indicated in brackets).
