# Supplementary material for: Veterinary antimicrobials practices for swine mycoplasmoses: a field survey and comparison with scientific and regulatory recommendations
Source: Porcine Health Manag. 2026 Jun 22;12:50. doi: 10.1186/s40813-026-00522-4 (PMC13595520; doi:10.1186/s40813-026-00522-4)
Supplement: Supplementary file 3 [file 40813_2026_522_MOESM1_ESM.docx]

*Supplementary Figure 1: Webpage appearance of the questionnaire*
